# Single Source Thermal Evaporation of Two-dimensional Perovskite Thin Films for Photovoltaic Applications

**DOI:** 10.1038/s41598-019-53609-0

**Published:** 2019-11-22

**Authors:** Zhuang-Hao Zheng, Hua-Bin Lan, Zheng-Hua Su, Huan-Xin Peng, Jing-Ting Luo, Guang-Xing Liang, Ping Fan

**Affiliations:** 1 Shenzhen Key Laboratory of Advanced Thin Films and Applications, College of Physics and Optoelectronic Engineering, Shenzhen University, Shenzhen, 518060 P.R. China; 2 College of Engineering Physics, Shenzhen Technology University, Shenzhen, 518118 P.R. China

**Keywords:** Solar cells, Materials for devices

## Abstract

Hybrid two-dimensional (2D) halide perovskites has been widely studied due to its potential application for high performance perovskite solar cells. Understanding the relationship between microstructural and opto-electronic properties is very important for fabricating high-performance 2D perovskite solar cell. In this work, the effect of solvent annealing on grain growth was investigated to enhance the efficiency of photovoltaic devices with 2D perovskite films based on (BA)_2_(MA)_3_Pb_4_I_13_ prepared by single-source thermal evaporation. Results show that solvent annealing with the introduction of solvent vapor can effectively enhance the crystallization of the (BA)_2_(MA)_3_Pb_4_I_13_ thin films and produce denser, larger-crystal grains. The thin films also display a favorable band gap of 1.896 eV, which benefits for increasing the charge-diffusion lengths. The solvent-annealed (BA)_2_(MA)_3_Pb_4_I_13_ thin-film solar cell prepared by single-source thermal evaporation shows an efficiency range of 2.54–4.67%. Thus, the proposed method can be used to prepare efficient large-area 2D perovskite solar cells.

## Introduction

Solar energy is a green clean energy source with a wide range of applications. Organic–inorganic hybrid perovskite solar cells have drawn great attention thanks to their high photoelectric conversion efficiency, simple manufacturing, and low cost^1–3^. The initial efficiency of perovskite solar cells was only 3.8%, but recently, the efficiency of single-junction perovskite solar cells has reached a high record to 24.2%^4^ and the perovskite/silicon tandem solar cell incresed to 25.2%^5^.

The device structures, recombination mechanisms, interface engineering, and material synthesis should be studied to further improve the efficiency. The key to improving the efficiency of solar cells is to improve the quality of perovskite film^6^. Solvent annealing is reportedly as an effective way to improve the crystallinity of some organic semiconductors, but this method’s effectiveness in inorganic semiconductors has not yet been confirmed. The current study shows that solvent annealing can be applied to increase the crystallinity and grain size of the perovskite films. Huang *et al*. found that the introduction of DMF/DMSO solvent vapor during the growth of CH_3_NH_3_PbI_3_ crystals can effectively improve the crystallinity and grain size of the film, passivate the film defects, and improve the device performance^7^. Liu *et al*. reported the introduction an anti-solvent vapor (e.g., alcohol vapor) to replace DMF vapor during the annealing procedure, which can improve the growth of perovskite crystals and increase the grain size of the perovskite MAPbI_3_ crystals, thus high crystallinity and pinhole-free MAPbI_3_ film could be obtained^8^. Zhang *et al*. created a different solvent atmosphere to anneal the perovskite film, the results show that the perovskite crystal quality was significantly improved when annealing in a poor mixed solvent [IPA: DMF = 100:1 (v/v)]^9^.

In addition to efficiency, the environmental stability and light stability under operating conditions are other key factors in photovoltaic and other optoelectronic applications^10^. Compared with its 3D counterpart, the Ruddlesden–Popper phase layered 2D perovskite thin film shows good stability but low efficiency^11,12^. The poor efficiency could be attributed to the inhibition of the out-of-plane charge transport of organic cations, which acted like insulating spacers between conductive inorganic plates^13^. Recently, attempts to utilize two-dimensional layered hybrid compounds in perovskite films have achieved breakthrough results. Smith I.C. *et al*. reported a layered (PEA)_2_(CH_3_NH_3_)_2_Pb_3_I_10_ perovskite light absorber for solar cell applications. The solar cell has an interesting open circuit voltage of 1.18 V and a photoelectric conversion efficiency of 4.73%. Moreover, this absorber was relatively stable up to 46 days in air with 52% relative humidity^14,15^. Mitzi D.B. explored the photovoltaic-related properties of 2D MA_2_Pb (SCN)_2_I_2_ perovskite, which can be used as an absorber layer for the top cell of a tandem solar cell^16^. Although superior device performance has not yet been achieved, this 2D layered mixtures have been demonstrated as effective new perovskite film with adjustable photoelectric properties and enhanced air stability^17,18^. In contrast to 3D perovskite, 2D perovskite [CH_3_(CH_2_)_3_NH_3_)_2_(CH_3_NH_3_)_n-1_Pb_n_I_3n+1_ (BA)_2_(MA)_n-1_Pb_n_I_3n+1_, n = 1, 2, 3, 4, …, ∞] have better optoelectronic property tunability because of their greater degree of freedom in quantum mechanics and chemistry, and, more importantly, higher environmental stability. Therefore, the development of 2D perovskite thin films will directly aid in improving the stability of perovskite solar cells.

Based on vacuum preparation method, the dual-source or single-source thermal evaporation methods, are also available to deposit perovskite thin films^19–21^. However, the dual-source thermal evaporation requires precise simultaneous control of the evaporation source of organic and inorganic materials, but the effective control of the film compounding process is very difficult. The easy deviation from the stoichiometric ratio directly leads to a decrease in film quality and repeatability^22^. To our knowledge, single-source thermal evaporation is a effective method for preparing large-area, high-efficiency perovskite solar cells^23^. In this study, 2D perovskite (BA)_2_(MA)_3_Pb_4_I_13_ thin film was prepared by single-source thermal evaporation, and the effects of solvent annealing on the microstructural and optoelectronic properties of the thin film were investigated.

## Results

**Figure 1:**
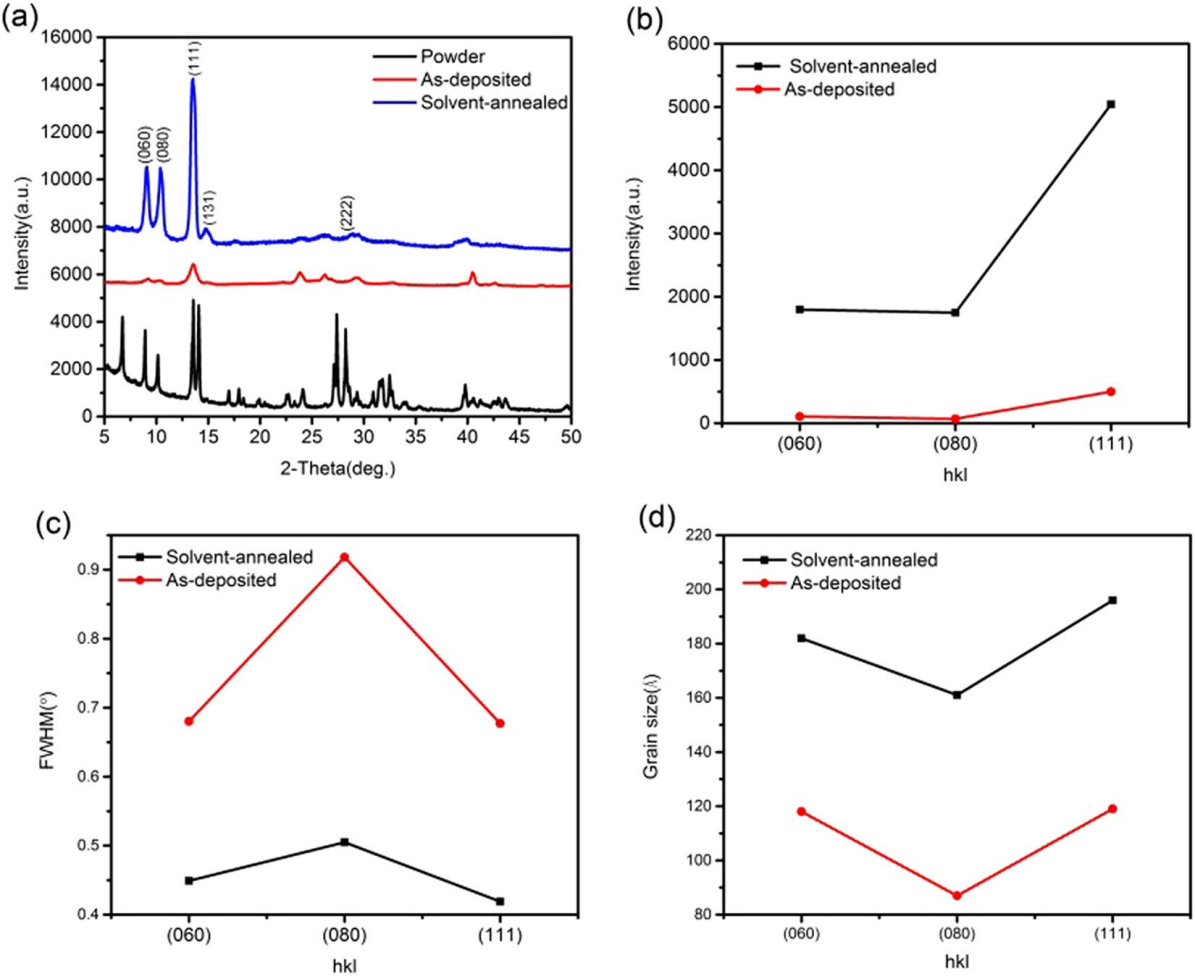
XRD patterns of the (**a**) (BA)_2_(MA)_3_Pb_4_I_13_thin films prepared by single-source thermal evaporation, (**b**) changes in the typical peak intensities, (**c**) FWHM changes, and (**c**) change in grain size.

Figure 1a shows the crystal structure of the (BA)_2_(MA)_3_Pb_4_I_13_ powder and the thin films including the as-deposited and solvent-annealed thin films. The prepared (BA)_2_(MA)_3_Pb_4_I_13_ powders have characteristic diffraction peaks of 2D perovskite. However, the as-deposited thin film shows broad peaks, which indicates low crystallinity. After the solvent annealing, the stronger characteristic diffraction peaks of the (060), (080), (111), (131), and (222) planes usually refer to the 2D (BA)_2_(MA)_3_Pb_4_I_13_ perovskite crystal structure. These results indicate that the perovskite crystallinity is increased, with fewer low-dimensional defects and/or larger perovskite grain sizes, and less scattering of internal grain boundaries (Fig. 1b)^24–27^. Figure 1c displays the FWHM of the 2D perovskite (060), (080), and (111) peaks. The FWHM of the solvent-annealed thin film is significantly smaller, indicating better crystallization^28^. Based on the Debye-Scherrer formula, *D* = *Kλ*/(*β* cos*θ*), (*D* is the grain size of crystals, *K* is a constant, *λ*is the wavelength of the X-ray, *β*is the FWHM, and *θ* is the diffraction angle^29^), the grain sizes of the as-prepared and solvent-annealed thin films were calculated and the results are shown in Fig. 1d. After solvent annealing, the 2D perovskite grain size becomes markedly larger, suggesting that the solvent annealing can improve the crystallinity of the thin film, which might lead to a higher efficiency for device.

**Figure 2:**
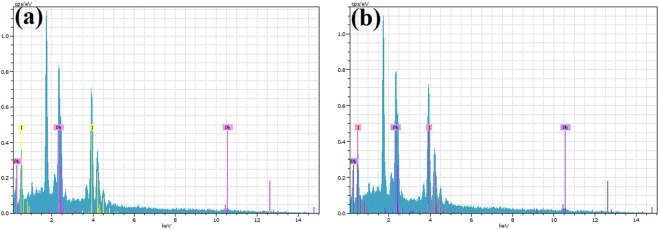
The EDS spectral line patterns of the (**a**) as-deposited and (**b**) solvent-annealed films.

**Figure 3:**
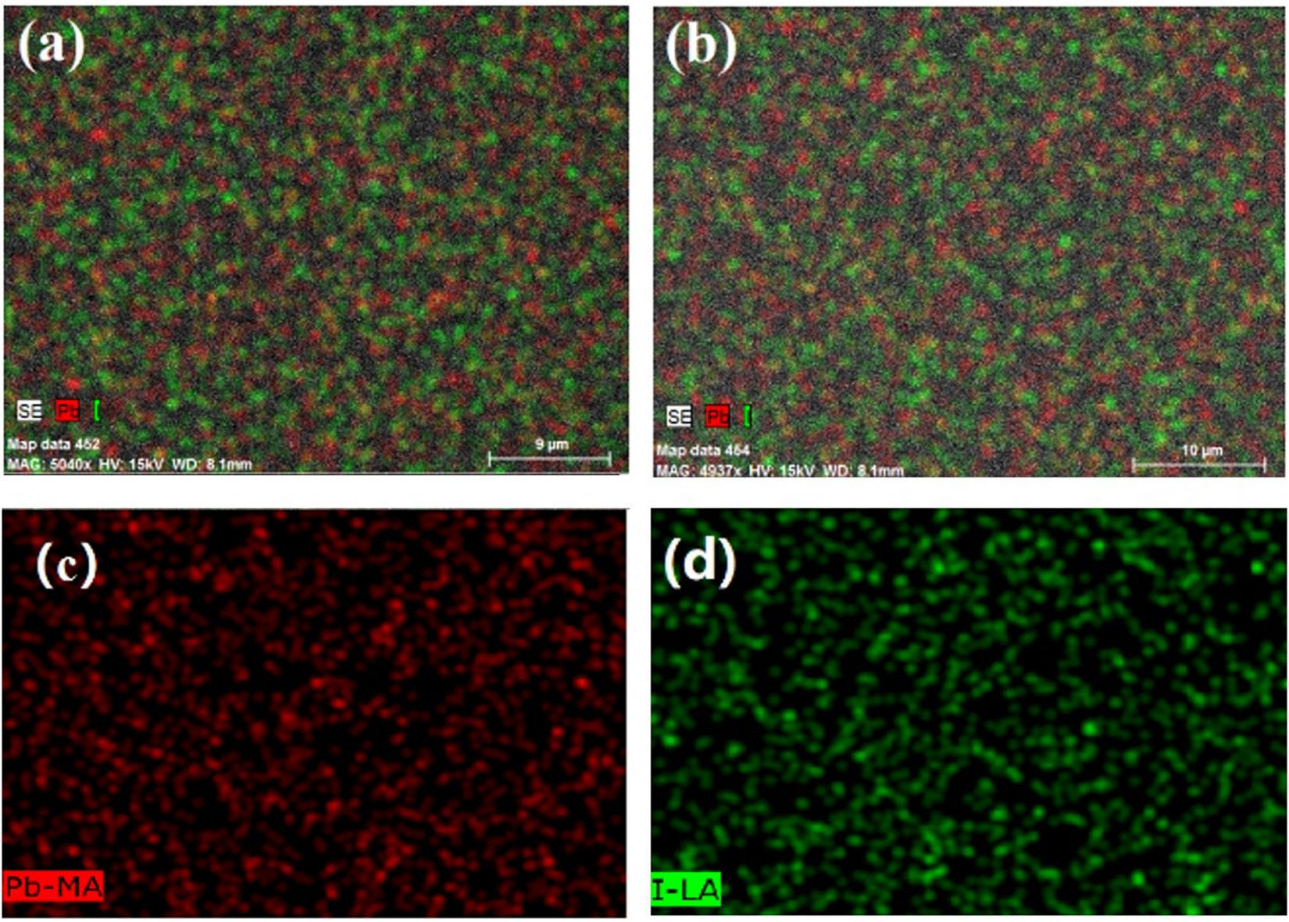
EDS-MAP of the (BA)_2_(MA)_3_Pb_4_I_13_ thin films (**a**) before and (**b**) after solvent annealing; distribution of (**c**) I and (**d**) Pb elements.

The composition of the (BA)_2_(MA)_3_Pb_4_I_13_ film is an important factor affecting the structural, electrical, and optical properties of the light-absorber. Figure 2 and Table 1 show the composition of the (BA)_2_(MA)_3_Pb_4_I_13_ thin films measured by EDS. Two typical peaks located at 2.48 and 3.98 keV, corresponding to the Pb and I elements. The atomic ratio of Pb to I of the as-deposited thin film is approximately 0.392. It decreases to 0.365 for the solvent annealed films, which is much close to the stoichiometry of the (BA)_2_(MA)_3_Pb_4_I_13_ film, indicating the formation of pure-phase 2D perovskite thin films. Figure 3 displays the elemental distribution of the thin film after solvent annealing, and shows that the Pb and I have uniform distribution in the entire plane without element enrichment or deficiency.

**Table 1:** Compositions of the as-deposited and solvent-annealed films determined by EDS analysis.

Sample	Pb (at%)	I (at%)	Pb/I
As-deposited films	28.21	71.79	0.392
Solvent annealed films	26.76	73.24	0.365

**Figure 4:**
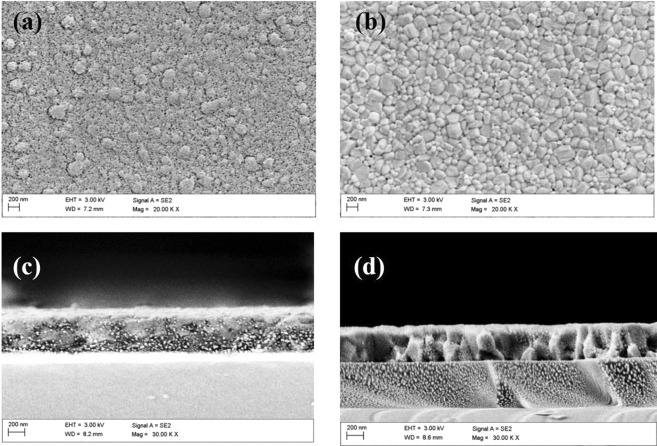
Morphology of the (**a**) as-deposited and (**b**) solvent-annealed film (**b**). Cross-sectional scanning electron micrographs of the (**a**) as-deposited and (**d**) solvent-annealed films.

Figure 4 show the morphology of as-deposited and solvent-annealed (BA)_2_(MA)_3_Pb_4_I_13_ thin films. Figure 4a illustrates that the as-deposited thin film exhibits complete surface coverage but with small grains on the substrate. The cross-section in the inset of Fig. 4a shows no distinct grains which may easily lead to poor reproducibility and photocurrent hysteresis of the 2D perovskite solar cells^30–33^. Solvent vapor of γ-butyrolactone introduced during the annealing of the 2D perovskite causes the recrystallization of (BA)_2_(MA)_3_Pb_4_I_13_. Precise control of recrystallization can improve the quality of perovskite film^34–36^. After the γ-butyrolactone vapor annealing treatment (Fig. 4b), the (BA)_2_(MA)_3_Pb_4_I_13_ thin film shows denser and larger grain distribution, and the defects are significantly reduced. Therefore, more photogenerated charges can successfully reach the electrode instead of recombining in the grain boundary.

**Figure 5:**
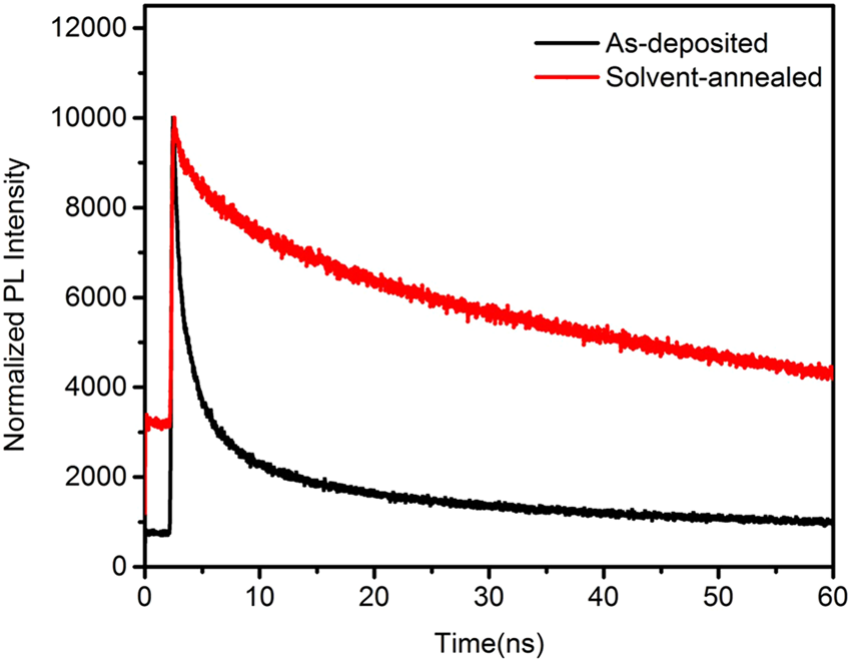
Time-resolved photoluminescence lifetime of the as-deposited and solvent-annealed (BA)_2_(MA)_3_Pb_4_I_13_ thin films.

Time-resolved PL (TRPL) decay measurements were performed to study the influence of the charge transfer process in the (BA)_2_(MA)_3_Pb_4_I_13_ thin film. Figure 5 displays the patterns and the lifetimes of the charge carriers in the thin films were estimated by fitting the data^37^. The average decay time (τ_ave_) of the (BA)_2_(MA)_3_Pb_4_I_13_ films were calculated according to the formula, τ_a_ = (A_1_τ_1_^2^ + A_2_τ_2_^2^)/(A_1_τ_1_ + A_2_τ_2_), and the charge carrier life time extracted from the as-deposited (BA)_2_(MA)_3_Pb_4_I_13_thin films is 1.34 ns^38^. Under γ-butyrolactone solvent annealing, the TRPL lifetimes are increased to 26.29 ns, which is in very good agreement with previously reported values^39^. Using the formula ${{L}_{D}}^{2}={\rm{D}}{\tau }_{s}$, in which the fluorescence lifetime of perovskites and the diffusion coefficients of electrons and holes D, the electron and hole diffusion lengths are deduced to be 314 nm and 266 nm, respectively^40^. The longer lifetime (τ_s_) indicates an increased charge-diffusion length (L_D_) of the (BA)_2_(MA)_3_Pb_4_I_13_ thin films because of the better crystallization in the solvent-annealed thin films. This characteristic reduces the recombination of photoelectron–hole pairs.

**Figure 6:**
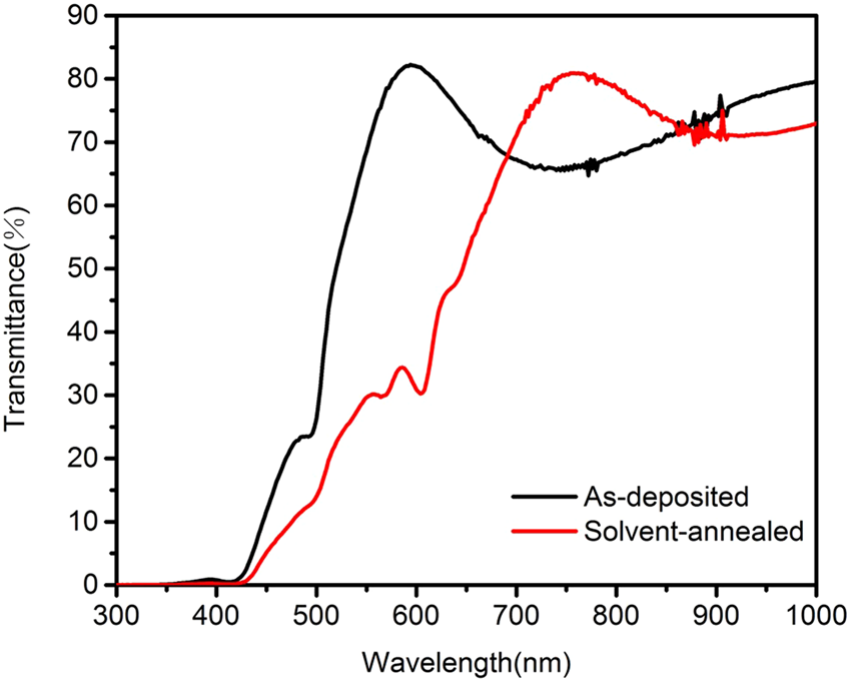
Optical transmittance spectra of the as-deposited and solvent-annealed (BA)_2_(MA)_3_Pb_4_I_13_ thin films.

**Figure 7:**
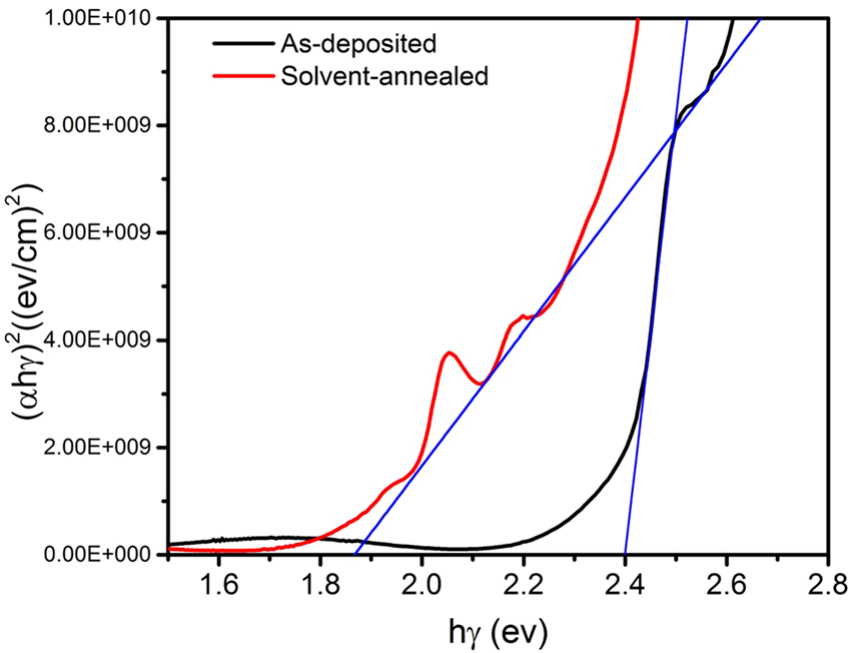
Estimation of the optical band gap of the as-deposited and solvent-annealed (BA)_2_(MA)_3_Pb_4_I_13_ thin films.

The optical transmittance properties were obtained by a UV/visible/near-IR spectrophotometer in the wavelength range of 300–1000 nm. Figure 6 shows the transmittance spectra for the as-deposited and solvent-annealed (BA)_2_(MA)_3_Pb_4_I_13_ thin films prepared by single-source thermal evaporation. As shown in the previous SEM image, more defect states due to smaller grains, the absorption edge is clearly moving toward the IR region after solvent annealing, indicating wide range of light absorption caused by enhanced crystallinity. The absorption range of the as-deposited thin film was lower than that of the annealed thin film due to the improvement of the film’s crystallinity as we mentioned above. Compared with perovskites with multiple nano-grains, the solvent-annealed perovskite film has fewer grain boundaries, this facilitates a greater range of light absorption by the absorbing layer. The band-gap energy can be calculated as $\alpha h\nu ={\rm{A}}{(h\nu -{E}_{g})}^{n}$, where *α* is the absorption coefficient, *hv* is the photon energy, *A* is the constant, *n* depends on the nature of transition, and *E* is the band-gap energy^41^. Figure 7 shows that the band gap of the as-deposited thin film is 2.40 eV and decreases to 1.89 eV after solvent annealing, which is close to the theoretical value^42^. Hydrogen bond exists in perovskite, the presence of hydrogen bonds may affect the optical band gap of (BA)_2_(MA)_3_Pb_4_I_13_, Similarly, Filip *et al*.^43,44^ have experimentally shown perovskite tunable optical bandgaps.

**Figure 8:**
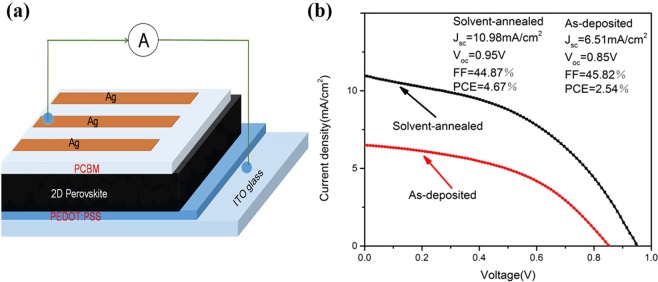
Device structure of (**a**) 2D perovskite solar cell; (**b**) J-V characteristics before and after solvent annealing.

The perovskite solar cells with a device structure of ITO/PEDOT: PSS/2D perovskite (BA)_2_(MA)_3_Pb_4_I_13_/PC_61_BM/Ag (Fig. 8a) were fabricated. PEDOT: PSS and PCBM were the hole and electron transport layers, respectively. Figure 8b shows the *J-V* curves of the (BA)_2_(MA)_3_Pb_4_I_13_ perovskite solar cells based on the as-deposited and solvent-annealed (BA)_2_(MA)_3_Pb_4_I_13_ thin film. The *J*_*sc*_, *V*_*oc*_, *FF*, *PCE*, *R*_*s*_, and *R*_*sh*_ of the corresponding devices are summarized in Table 2. In the 2D perovskite solar cell, the (BA)_2_(MA)_3_Pb_4_I_13_ thin films without γ-butyrolactone vapor treatment are presented as the as-deposited PSCs, while the films treated with γ-butyrolactone are presented as the solvent-annealed PSCs. The as-deposited PSCs exhibit *J*_*sc*_ of 6.51 mA/cm^2^, *V*_*OC*_ of 0.85 V, *FF* of 45.82%, *R*_*s*_ of 729.93 Ω, and *R*_*sh*_ of 616.78 Ω. These characteristics result in a low PCE of 2.54%. Compared with the as-deposited PSCs, when the γ-butyrolactone solvent vapor is introduced during annealing, the performance of the 2D perovskite solar cell is significantly enhanced. *J*_*sc*_ substantially increases to 10.98 mA/cm^2^, *V*_*oc*_ increases to 0.95 V, *R*_*s*_ is reduced to 26.55 Ω, and PCE increases to 4.67%. The γ-butyrolactone vapor during annealing can lead to enhanced crystallinity and larger grain size of (BA)_2_(MA)_3_Pb_4_I_13_, passivate defects, and improve device performance. Hence, the solvent annealing produces a high quality non-porous 2D perovskite film with a high purity phase, complete surface coverage, and good crystallinity. These characteristics can suppress internal recombination and leakage currents and promote photoelectric conversion of 2D perovskite solar cells.

**Table 2:** Photovoltaic performances and fitting parameters used for the impedance spectra of the (BA)_2_(MA)_3_Pb_4_I_13_-based perovskite solar cells before and after solvent annealing.

Sample	V_oc_(V)	J_sc_(mA/cm^2^)	FF	PCE	R_s_(Ω)	R_sh_(Ω)
As-deposited	0.85	6.51	45.82%	2.54%	729.93	616.78
Solvent-annealed	0.95	10.98	44.87%	4.67%	26.55	128.70

## Conclusions

The effect of solvent annealing on grain growth is investigated to enhance the photovoltaic-device efficiency of 2D perovskite (BA)_2_(MA)_3_Pb_4_I_13_ thin film prepared by single-source thermal evaporation. Solvent annealing can effectively enhance the crystallization of (BA)_2_(MA)_3_Pb_4_I_13_ thin film with denser and larger crystal grains. The element ratio of Pb/I is close to the ideal stoichiometric ratio. The films show a favorable band gap of 1.896 eV and long electron and hole diffusion lengths of 314 nm and 266 nm, respectively. The performance of the (BA)_2_(MA)_3_Pb_4_I_13_ perovskite solar cell is significantly enhanced, that is, *J*_*sc*_ remarkably increases to 10.98 mA/cm^2^, *V*_*oc*_ increases to 0.95 V, and *R*_*s*_ is reduced to 26.55 Ω. The solvent-annealed (BA)_2_(MA)_3_Pb_4_I_13_thin-film solar cell prepared by single-source thermal evaporation shows an efficiency of 4.67%. Thus, the proposed method is promising for preparing large-area and efficient 2D perovskite solar cells.

## Methods

### (BA)_2_(MA)_3_Pb_4_I_13_ crystal and powder preparation

**Figure 9:**
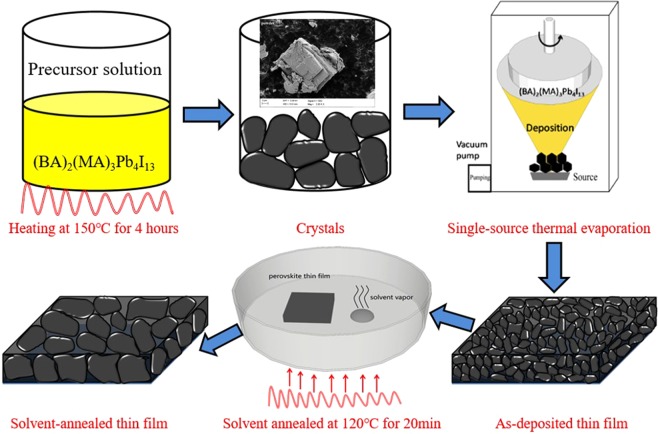
Schematic of the fabrication procedure including crystal preparation, single-source thermal evaporation, and solvent annealing.

PbI_2_ (7.38 g, 99.99%, Xi’an Polymer Light Technology), MAI (1.91 g, 99.5%, Xi’an Polymer Light Technology), and BAI (1.61 g, 99.5%, Xi’an Polymer Light Technology) were mixed in γ-butyrolactone (150 ml, 99%, TCI) in the beaker for 24 h with constant magnetic stirring. The 2D perovskite solution was then transferred onto a glass culture dish and maintained at 150 °C on a hot plate. Until all 2D perovskite solutions were evaporated, (BA)_2_(MA)_3_Pb_4_I_13_ crystals can be obtained as shown in Fig. 9. Then, the prepared (BA)_2_(MA)_3_Pb_4_I_13_ crystals were ground into powders as the film evaporation material.

### (BA)_2_(MA)_3_Pb_4_I_13_ thin-film preparation

Figure 9 shows the fabrication of the crystals, including the (BA)_2_(MA)_3_Pb_4_I_13_ crystals preparation, single-source thermal evaporation, and solvent annealing. Prior to deposition, the ITO glass substrate was cleaned, and 0.8 g of (BA)_2_(MA)_3_Pb_4_I_13_ perovskite powder was weighed. The powder was placed in the evaporation boat. The distance from the evaporation source to the substrate was 25 cm, and the substrate speed was 40 rpm. Once the chamber pressure was pumped down to below 1 × 10^−3^ Pa, the working current of the evaporation source was rapidly raised to 150 A, and then the film was deposited. Until the powder was completely evaporated, the as-deposited 2D perovskite (BA)_2_(MA)_3_Pb_4_I_13_ thin films has a thickness of approximately 400 nm. Solvent annealing was then performed under a γ-butyrolactone atmosphere (40 μl) at 120 °C for 20 min.

### Device fabrication

The perovskite solar cells have a device structure of ITO/PEDOT: PSS/(BA)_2_(MA)_3_Pb_4_I_13_/PC_61_BM/Ag was prepared. An aqueous solution of PEDOT-PSS (CLEVIOS PVP AI4083) was spin-coated onto ITO glass substrate to form a 50-nm thick thin film (4500 rpm for 40 s). The obtained PEDOT-PSS film was placed on a hot plate at 160 °C for 20 minutes and then transferred to a single source evaporation deposition system. The (BA)_2_(MA)_3_Pb_4_I_13_ absorber layer was deposited by single-source thermal evaporation and then solvent annealed in a N_2_-filled glove-box for 20 min at 120 °C. The PC_61_BM solution (20 mg/ml in chlorobenzene) was spin-coated on the (BA)_2_(MA)_3_Pb_4_I_13_ thin film at 3000 rpm for 30 s. Lastly, 90 nm-thick Ag cathode was prepared by thermal evaporation in a vacuum of approximately 3.0 × 10^−4^ Pa.

### Characterization

The crystalline structure of the (BA)_2_(MA)_3_Pb_4_I_13_ thin films and powder were analyzed by X-ray diffractometer (Ultima IV). The composition and surface morphology of perovskite film and powder were analyzed by energy-dispersive X-ray microanalysis system (Bruker QUANTAX 200, Bruker, Billerica, MA, USA) and a SUPRA 55 scanning electron microscope, respectively. The time-resolved PL was recorded using the steady-state spectroscopy and time-resolved (Fluo Time 300, Pico Quant GmbH). The thickness of the (BA)_2_(MA)_3_Pb_4_I_13_ thin films were measured by a DEKTAK XT profilometer (Bruker, Billerica, M A, USA). The optical transmittance properties were obtained by a UV/visible/near-IR spectrophotometer (Lambda 950, PerkinElmer). The *J-V* curves of the 2D perovskite solar cells were recorded in simulated AM 1.5 G conditions (100 mW/cm^2^) with a Keithley 2400 Source Measure Unit.

## Data Availability

All data included in this study are available upon request by contact with the corresponding author.
